# Protein or Amino Acid Intake at Breakfast for Muscle Mass Gain in Older Adults: A Systematic Review and Meta‐Analysis

**DOI:** 10.1002/fsn3.72171

**Published:** 2026-07-24

**Authors:** Takashi Ikeda, Naonori Tashiro, Hiroyuki Ohtsuka, Noyuri Yamaji, Masaaki Matoba, Hisashi Noma, Erika Ota, Takeshi Hasegawa

**Affiliations:** ^1^ Medical School, Department of Health Sciences, Major in Rehabilitation Science, Physical Therapy Program Nagoya City University Nagoya city Japan; ^2^ Institute of Clinical Epidemiology Showa Medical University Tokyo Japan; ^3^ School of Nursing and Rehabilitation Sciences Showa Medical University Yokohama Japan; ^4^ Department of Physical Therapy, School of Rehabilitation Gunma Paz University Gunma Japan; ^5^ Corporate Development Office Showa Medical University Shinagawa‐ku, Tokyo Japan; ^6^ Department of Interdisciplinary Statistical Mathematics The Institute of Statistical Mathematics Tokyo Japan; ^7^ Graduate School of Nursing Science, Global Health Nursing St Luke's International University Tokyo Japan; ^8^ Division of Nephrology, Department of Medicine Showa Medical University School of Medicine Tokyo Japan; ^9^ Department of Hygiene, Public Health and Preventive Medicine Showa Medical University School of Medicine Tokyo Japan; ^10^ Center for Innovative Research for Communities and Clinical Excellence Fukushima Medical University Fukushima Japan

**Keywords:** branched chain amino acids, breakfast, decreased dietary intake, protein

## Abstract

Preventing sarcopenia is a major goal for older adults. High protein breakfast, while maintaining overall daily intake, has been proposed as a promising approach to support muscle health. However, the optimal timing of protein consumption remains uncertain. Therefore, the specific effects of breakfast protein intake are therefore important for refining nutritional recommendations. The methodology of this review is as follows: which included randomized controlled trials (RCTs) comparing morning protein intake with no intake in healthy and frail adults aged ≥ 60 years. A trained librarian developed comprehensive search strategies for MEDLINE, CENTRAL, EMBASE, and ClinicalTrials.gov. Two reviewers independently screened the studies, extracted data, and assessed the risk of bias using Cochrane's RoB tool 1.0. Standardized mean differences (SMD) were calculated using Review Manager. Seven RCTs involving 305 participants were included. Five studies (*n* = 181) reported lean mass with an SMD of −0.11 (95% CI, −0.41 to 0.18). Six studies (*n* = 274) measured grip strength (SMD −0.01; 95% CI, −0.25 to 0.23). Three studies (*n* = 157) evaluated lower limb strength (SMD −0.11; 95% CI, −0.41 to 0.18), showing similar results. Morning protein intake had a marginal reduction in total daily energy intake (SMD −0.35; 95% CI, −0.70 to 0.00), and had a significant reduction in trials without exercise (SMD, −0.47; 95% CI, −0.82 to −0.11). As a conclusion, morning protein intake may not improve muscle mass, strength, or physical performance among older adults and may reduce little food intake without exercise. But the evidence is very uncertain. Further high‐quality RCTs are needed for the effects of morning protein intake on muscle health in older adults.

## Graphical abstract

Preventing sarcopenia is a major goal for older adults. High protein has been proposed as a promising approach to support muscle health. However, the optimal timing of protein consumption remains uncertain. Therefore, the specific effects of breakfast protein intake are important for refining nutritional recommendations. Morning protein intake may not improve muscle mass, strength, or physical performance among older adults and may reduce little food intake without exercise. But the evidence is very uncertain.

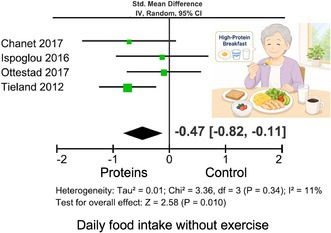

**Abbreviations**
BIA: bioelectrical impedance method
CENTRAL: Cochrane Central Register of Controlled Trials
ITT: intention to treat
MPS: muscle protein synthesis
PRISMA: Preferred Reporting Items for Systematic Reviews and Meta‐Analyses
PROSPERO: Prospective Register of Systematic Reviews
RCTs: randomized controlled trials
RevMan: Review Manager
RoB: risk of bias
SMD: standardized mean difference

## 1 Background

Sarcopenia and frailty affect the health of community‐dwelling older adults (Roberts et al. 2021). The mortality of older inpatients worsens as sarcopenia elements and malnutrition increase (Gümüşsoy et al. 2021). Consequently, clinical nutrition societies recommend sufficient protein intake to support muscle mass gain (Roberts et al. 2021). The recommended daily protein intake is 1.0–1.2 g/kg body weight (BW)/day for healthy older adults and 1.2–1.5 g/kg BW/day for those at risk of malnutrition (Deutz et al. 2014). The Japanese Dietary Intake Standards 2020 (Ministry of Health, Labour and Welfare 2020) recommend a targeted daily protein consumption of 90–120 g for men and 69–93 g for women aged 65–74 years with standard living intensity. However, 10% of community‐dwelling older adults and 35% of nursing home residents do not achieve the recommendations (Tieland et al. 2012).

For muscle mass gain, both the timing and amount of protein intake are critical (Gorissen et al. 2015; Yasuda et al. 2022). Traditionally, protein consumption is recommended immediately after exercise, consistent with the International Society of Sports Nutrition statement for athletes and healthy individuals (Jäger et al. 2017), which recommends intake immediately before or after exercise. Maximized muscle protein synthesis (MPS) can be induced by pre‐exercise protein consumption without additional improvements in performances (Jäger et al. 2017). Carbohydrate‐based protein consumption after exercise drives muscle glycogen recovery and mitigates muscle damage (Jäger et al. 2017). In contrast, MPS can be maintained for 24 h at a relatively constant level if protein or amino acids are consumed throughout the day, regardless of exercise (Burd et al. 2011; Ra et al. 2018). These contrasting findings (Burd et al. 2011; Ra et al. 2018) indicate that protein consumption timing may be flexible for individuals who exercise at the same time daily.

The optimal timing of protein consumption in healthy adults remains inconclusive (Wirth et al. 2020). However, dietary diversity exists in terms of the amount and number of meals consumed. The interval between meals is typically longest between supper and the next breakfast, often resulting in lower protein intake at breakfast (Aoyama et al. 2021). Equalizing protein consumption across three meals may be more effective for MPS than skewing intake toward the evening meal (Mamerow et al. 2014). Another study (Chanet et al. 2017) reported that consuming protein at breakfast for 6 weeks augments postprandial MPS and muscle mass gain, even without exercise, by supporting sustained serum amino acid concentrations. These results suggest that protein weighting at breakfast may effectively promote muscle mass gain, potentially compensating for dietary imbalance, influencing postprandial metabolism, and accelerating MPS (Pekmez et al. 2020; Ottestad et al. 2017).

Clinical trials (Ra et al. 2018; Mamerow et al. 2014; Chanet et al. 2017; Ottestad et al. 2017; Norton et al. 2016; Ispoglou et al. 2016) have searched for desirable protein consumption timings, including pre‐exercise, breakfast, and pre‐sleep, in addition to the traditional standard. Although these trials reported benefits for muscle mass or strength at various timings (Wirth et al. 2020), the most desirable timing remains inconclusive due to differences in consumption protocols (with or without exercise) (Ra et al. 2018; Mamerow et al. 2014; Chanet et al. 2017; Ottestad et al. 2017; Norton et al. 2016; Ispoglou et al. 2016).

Because breakfast typically contains lower protein amounts (Aoyama et al. 2021), which, along with previous research (Mamerow et al. 2014; Chanet et al. 2017), led to the hypothesis that protein weighting at breakfast may improve muscle mass and strength in older adults (Oktaviana et al. 2020; Liao et al. 2018; Dedeyne et al. 2017; Finger et al. 2015). Systematic reviews have shown that combining exercise with protein intake improves muscle mass and strength in older adults. Although increasing protein intake at breakfast without reducing intake at other meals has been proposed as an efficient way to maximize MPS, its effectiveness in older adults remains unclear.

## 2 Methods

### 2.1 Study Design and Objectives

This study was a systematic review and meta‐analysis that aimed to evaluate the benefits and harms of protein or amino acid intake at breakfast in older adults. We assessed the effects on lean muscle mass and physical function compared with no intervention or placebo. The review was reported following the PRISMA guidelines (Page et al. 2021). This systematic review was registered with PROSPERO registration ID: CRD42022298204 (https://www.crd.york.ac.uk/PROSPERO/display_record.php?RecordID=298204), and its protocol paper (Ikeda et al. 2024) was published in Feb 2024.

### 2.2 Eligibility Criteria

#### 2.2.1 Types of Studies

The following study types were included: 1. randomized controlled trials (RCTs) and crossover RCTs (only the pre‐arm if the washout period was not stated); 2. Peer‐reviewed papers in English, and were excluded: 1. observational studies and case reports; 2. RCTs without control groups; 3. non‐RCTs with intervention; and 4. quasi‐RCTs.

#### 2.2.2 Population

The population included healthy or frail older adults aged 60 years or older, with no restrictions on race or sex. Although frailty with a possibility overlapping sarcopenia, frailty does not indicate just muscle mass deficit. It widely indicates delicate conditions such as physical function, psychological conditions, and social network (Rockwood et al. 2005). Therefore, we excluded trials focused on sarcopenia or specific diseases.

#### 2.2.3 Types of Interventions

The intervention period was 1 month or longer, consisting of protein or amino acid consumption at breakfast, with and without exercise, compared with no intervention or placebo. The type of protein—whether animal (e.g., whey) or plant‐based (e.g., soy)—was not restricted. We excluded the trials focused on proteins, including amino acid ingestion with meals other than breakfast.

#### 2.2.4 Critical Outcomes

Critical outcomes were muscle mass (total lean mass and appendicular lean mass) and muscle strength (grip strength, knee extensor strength, and leg press). If the units of evaluation differed across studies, the standardized mean difference (SMD) was used as an effect measure. Critical outcomes were compared separately.

#### 2.2.5 Important Outcomes

Important outcomes included:

1. Physical performance tests (e.g., sit‐to‐stand test, balance test)
2. Gait speed
3. Harms (decreased dietary intake and deterioration of renal function)

### 2.3 Electronic Searches

The following electronic databases were searched for possible studies: MEDLINE, Cochrane Central Register of Controlled Trials (CENTRAL), EMBASE, and ClinicalTrials.gov. The electronic search was conducted from 22 Feb 2024. The search strategy was designed for MEDLINE and was appropriate for CENTRAL, EMBASE, and ClinicalTrials.gov. The librarians who designed the search strategy for this systematic review used Boolean search logic.

Keywords for search as follows: search: “aged”, “elder”, “older”, “old‐adult”, “old‐age”, “geriatr”, “senil”, and “senior”; “protein”, “proteins”, “amino acids”, “BCAA”, “aminoisobutyric acid”, “alloisoleucine”, “valine”, and “lysine”; and “breakfast”, and “morning”.

The entire search strategies and details are shown in Table S1, Literature search strategy for each database.

### 2.4 Data Collection and Analysis

#### 2.4.1 Selection of Studies

**FIGURE 1:**
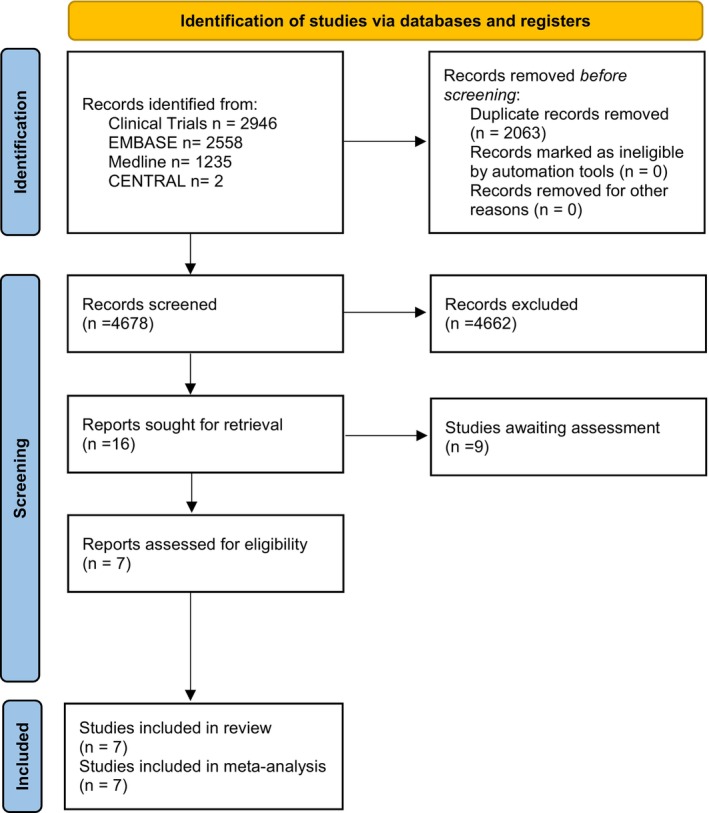
PRISMA 2020 flow diagram.

Four authors (TI, NT, HO, and MM) screened the titles and abstracts to obtain potential articles from electronic searches. Duplicate removal was conducted using Rayyan (n.d.). Two authors (TI and NT) independently reviewed the full text of the selected studies, which were refined in the initial search, and decided whether to include the studies for data extraction. Papers that met the exclusion criteria were excluded, and reasons were recorded using the Rayyan system. Disagreements regarding study inclusion for data extraction were resolved by consensus between the two authors and, if needed, consulted with the chief of Showa Medical University Institute of Clinical Epidemiology (TH). The search procedure and results are shown in the PRISMA flow diagram (Figure 1).

#### 2.4.2 Data Extraction and Management

For eligible studies, data extraction was conducted on basic information, study setting and design, participant characteristics, type of interventions or controls, relevant outcome measures and data, and the amount and reasons for missing data.

#### 2.4.3 RoB Assessment in Included Studies

Two authors (TI and NT) independently assessed the RoB of the included studies using Cochrane's RoB tool 1.0 for randomized controlled trials (National Institutes of Health 2011). The authors evaluated seven domains of RoB as follows: (1) sequence generation, (2) allocation concealment, (3) blinding of participants and personnel, (4) blinding of the outcome assessor, (5) incomplete outcome data, (6) selective outcome reporting, and (7) other biases (i.e., imbalance at baseline). Disagreements in the RoB between the two authors led to enquiries regarding third author (TH) coordination. In the above domains, the authors classified the included studies as having a low RoB, unclear, or high RoB.

#### 2.4.4 Measures of Treatment Effect

The authors adopted the mean difference for gait speed and the SMD for the other outcomes. Measuring treatment effects, 95% confidence intervals were obtained.

#### 2.4.5 Handling Missing Values

When there were missing values, drop out ratio 20% or higher, or an intention‐to‐treat (ITT) analysis design, a sensitivity analysis was conducted by Review Manager (RevMan) software version 5.4 (Cochrane Collaboration, Oxford, UK) for the effect of the listed paper. The authors made an enquiry to the corresponding author regarding missing data and the statistical treatment of the listed paper.

### 2.5 Synthesis Methods

The extracted data were pooled using the DerSimonian and Laird (Higgins et al. 2003) random‐effects model, and influential analysis for potential outlying studies were conducted using the methods of Viechtbauer and Cheung (DerSimonian and Laird 1986) and Noma (Viechtbauer and Cheung 2010). The integrated data were analyzed using RevMan to create summary tables (Noma 2020). Statistical heterogeneity between included studies was assessed using the heterogeneity variance *τ* (Gümüşsoy et al. 2021), Higgins' *I* ^2^ statistic, and Q statistic. *I* ^2^ values (Higgins et al. 2003) were interpreted as follows: 0%–40% may not be important; 30%–60% may represent moderate heterogeneity; 50%–90% may represent substantial heterogeneity; 75%–100% considerable heterogeneity. The authors describe the direction and magnitude of the effects and the degree of overlap of the confidence intervals in a forest plot table. Although we had planned to conduct subgroup analyses based on participants' health status (healthy vs. frail older adults), this was not performed as fewer than ten studies were included.

### 2.6 Assessment of Reporting Bias

We had planned to conduct assess publication bias (Egger et al. 1997) when the number of RCTs included in the meta‐analysis was > 10. This was not performed as fewer than ten studies were included.

### 2.7 Sensitivity Analysis

Studies with high RoB (e.g., unblinded allocation, open‐label design, or inappropriate outcome measures) showing inconsistencies were excluded from the analysis.

### 2.8 Sub‐Group Analysis

Subgroup analysis was conducted in accordance with the protocol paper (Ikeda et al. 2024), focusing on the presence or absence of frailty and the with or without of exercise interventions. If fewer than 10 trials were included, subgroup analyses were not performed. Instead, a sensitivity analysis was conducted.

### 2.9 Assessing Certainty of Evidence

Critical and important outcomes are presented as summaries of findings in accordance with the Cochrane Handbook (Cochrane Training 2022). The Grading of Recommendations, Assessment, Development, and Evaluation (GRADE) methodology (Guyatt et al. 2011) provided evidence for the critical outcomes. Each GRADE criterion and the overall certainty of evidence were independently graded from high to very low by three authors (TI, NY, and HO). Another author (TH) resolved discrepancies between the two authors when needed. Subgroup analyses were not conducted because fewer than ten studies were included.

## 3 Results

### 3.1 Search Results

The authors identified 4678 studies from the electronic searches after removing duplicates. We excluded 4662 identified studies based on title and abstract screening, leaving 16 studies for full‐text screening. Of these 16 studies, we excluded nine studies (Norton et al. 2016; Kim et al. 2021; Johnson et al. 2021; Mori et al. 2023; Park et al. 2023; Zeng et al. 2019; Bhasin et al. 2018; ClinicalTrials.gov 2022, 2018) due to assessment of study design, wrong comparator or interventions, outcomes, and ongoing studies. Therefore, seven RCTs were included in this review (Figure 1).

### 3.2 Characteristics of Included Studies

A total of 305 participants were included in the meta‐analyses of seven RCTs by de Azevedo Bach et al. (2022), Chanet et al. (2017), Ottestad et al. (2017), Ispoglou et al. (2016), Van Wymelbeke et al. (2016), Oesen et al. (2015), and Tieland et al. (2012) (Table 1). The critical outcomes were reported by five RCTs (181 participants, 94 in intervention groups, 87 in control groups) for lean mass, and six RCTs (274 participants, 156 in intervention groups, and 118 in control groups) for grip strength. All studies assessed the critical outcomes of lean mass and/or grip strength. Regarding adverse events, all studies reported on decreasing dietary intake; just one study (Ottestad et al. 2017) reported on renal function; therefore, the deterioration of renal function was not reviewed in this study.

**TABLE 1:** Description of all studies included.

Study settings	Total number of patients (*n*)	Subjects	Mean age	Duration	Intervention
Single‐center study	31	Resistance‐trained older persons	Intervention group, 66.9 Control group, 65.8	12 weeks	Intervention Group (*n* = 15), 20 g of whey protein in the morning and evening Control Group (*n* = 16), 20 g of maltodextrin in the morning and evening
Single‐center study	24	Healthy older men	Intervention group, 70.3 Control group, 70.8	6 weeks	Intervention group (*n* = 12), 20 g of whey protein, 3 g of total leucine, and 800 IU of vitamin D Control group (*n* = 12), a flavored watery placebo product
Single‐center study	36	Community dwelling older persons	Intervention group, 76.8 Control group, 77.1	12 weeks	Intervention Group (*n* = 17), 20 g of milk and whey protein in the morning and evening Control Group (*n* = 19), An isocaloric, non‐nitrogenous control drink
Single‐center study	25	Aged 65–75 years volunteers	Intervention group, 71.5 Control group, 71.8	12 weeks	Intervention Group (*n* = 16), 20 or 40% of leucine contained amino‐acid mixtures in the morning and evening Control Group (*n* = 8), An isocaloric, non‐nitrogenous control drink
Multi‐center study	68	Nursing home residents	Intervention group, 76.8 Control group, 77.1	12 weeks	Intervention Group (*n* = 46), 65 g of the brioche roll contained 12.8 g of protein or 200‐ml energy‐dense liquid contained 14 g of protein Control Group (*n* = 22), the usual breakfast
Multi‐center study	56	Retirement care facilities users	Intervention group, 81.8 Control group, 83	24 weeks	Intervention Group (*n* = 25), nutrient supplement drink contained 20.7 g of protein in the every morning and additionally, after training session twice a week Control Group (*n* = 26), cognitive training
Single‐center study	65	Frailty older persons	Intervention group, 83 Control group, 78	24 weeks	Intervention group (*n* = 34), 15 g of milk protein twice daily in the morning and lunch Control group (*n* = 31), placebo beverage containing no protein, 7.1 g lactose, and 0.4 g calcium

Abbreviation: RCT, randomized control trial.

### 3.3 RoB Assessment

The RoBs of the included RCTs are summarized in Table 2. One RCT (Van Wymelbeke et al. 2016) was graded as having a high risk of RoB due to lack of allocation concealment in the selection process. In this study, the allocation was opened to participants and assessors to determine whether interventions, which were resistance exercise training combined with or without nutrient supplementation or cognitive training, were the control group. The other six RCTs were moderate (Ispoglou et al. 2016; Oesen et al. 2015) and low RoB (Tieland et al. 2012; Chanet et al. 2017; Ottestad et al. 2017; de Azevedo Bach et al. 2022).

**TABLE 2:** Summary of risk of bias for primary outcomes in included studies.

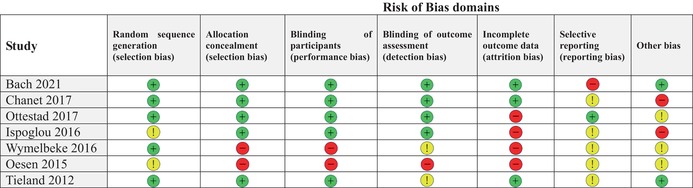

*Note:* Judgment: 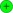, Low risk; 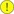, Unclear risk; 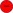, High risk.

### 3.4 Findings of Critical Outcome

#### 3.4.1 Muscle Strength (Grip Strength, Lower Limb Extensor Strength)

**FIGURE 2:**
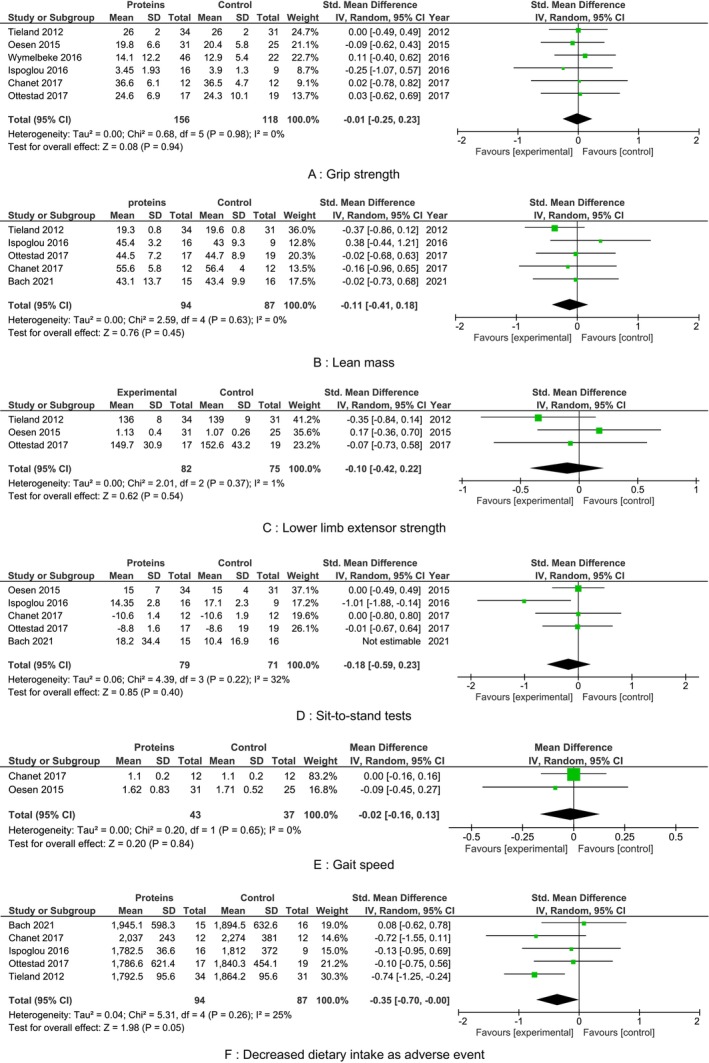
Forest plots for critical and important outcomes. Forest plot of proteins intake at breakfast and control group comparing the grip strength (A), lean mass (B), lower limb extensor strength (C), gait speed (D), sit‐to‐stand tests (E), decreased dietary intake as adverse event (F).

Data on grip strength were obtained from six studies (Tieland et al. 2012; Chanet et al. 2017; Ottestad et al. 2017; Ispoglou et al. 2016; Van Wymelbeke et al. 2016; Oesen et al. 2015). The 274 participants included in these studies were compared with the control group using the SMD (Table 3), showing a forest plot (Figure 2A). The SMD was −0.01 (*p* = 0.94; 95% CI, −0.25 to 0.23). No clear differences in protein intake were observed. No statistical heterogeneity was observed for grip strength (*I* ^2^ = 0%, *χ* ^2^ = 0.68, *p* = 0.98).

**TABLE 3:** Summary of findings.

Outcome	Relative effect (95% CI)	Absolute effects (95% CI)	Certainty
No of participants (studies)			
Grip strength No of participants: 274 (6 RCTs)	Not estimable	SMD 0.01 lower (0.25 lower to 0.23 higher)	⊕⊕⊕◯◯ Low^a^, ^b^
Lean mass No of participants: 181 (5 RCTs)	Not estimable	SMD 0.11 lower (0.41 lower to 0.18 higher)	⊕⊕◯◯ Low^c^, ^d^
Lower limb extensor strength No of participants: 157 (3 RCT)	Not estimable	SMD 0.1 lower (0.42 lower to 0.22 higher)	⊕◯◯◯ Very low^b^, ^d^, ^e^
Sit‐to‐stand tests No of participants: 150 (4 RCT)	Not estimable	MD 0.02 lower (0.16 lower to 0.13 higher)	⊕◯◯◯ Very low^b^, ^d^, ^f^
Gait speed No of participants: 80 (2 RCT)	Not estimable	SMD 0.18 lower (0.59 lower to 0.23 higher)	⊕◯◯◯ Very low^b^, ^d^, ^f^
Decreased dietary intake (adverse event) No of participants: 181 (5 RCT)	Not estimable	SMD 0.35 SD lower (0.7 lower to 0)	⊕◯◯◯ Very low^b^, ^d^, ^f^

Abbreviations: CI, confidence interval; MD, mean difference.

^a^ Most study protocols were not published, and two of six studies have high risk of bias.

^b^ The area of the CI straddles the zero of the standard.

^c^ Three of five studies have high rick of bias.

^d^ Fewer sample size.

^e^ A third of participants were open labeled.

^f^ Two of third for participants were open labeled.

Three studies (157 persons) provided data on lower limb extensor strength. Two studies tested knee extensor strength (Tieland et al. 2012; Oesen et al. 2015), the other one study (Ottestad et al. 2017) used the leg press test. The data were combined as the lower limb extensor strength and calculated for SMD (Table 3), showing a forest plot (Figure 2C). The SMD was −0.11 (*p* = 0.54; 95% CI, −0.41 to 0.18). Similar to lean mass and grip strength, no clear differences between the two groups were observed for protein intake. No statistical heterogeneity was observed for lower limb extensor strength (*I* ^2^ = 1%, *χ* ^2^ = 2.01, *p* = 0.37).

#### 3.4.2 Muscle Mass (Lean Mass)

Data on lean mass were available from five studies (Tieland et al. 2012; Chanet et al. 2017; Ottestad et al. 2017; Ispoglou et al. 2016; de Azevedo Bach et al. 2022). The 181 patients included in these studies were compared with the control group as SMD (Table 3), showing a forest plot (Figure 2B). The SMD was −0.11 (*p* = 0.45; 95% CI, −0.41 to 0.18). No clear differences between the two groups on protein intake were observed. No statistical heterogeneity was observed for the lean mass (*I* ^2^ = 0%, *χ* ^2^ = 2.59, *p* = 0.45).

### 3.5 Findings of Important Outcomes

#### 3.5.1 Physical Performance Test (Sit‐To‐Stand Tests)

Four studies (150 individuals) provided data on sit‐to‐stand tests as a physical performance test. Two studies used the 5‐times sit‐to‐stand test (Chanet et al. 2017; de Azevedo Bach et al. 2022), while the other studies (Ispoglou et al. 2016; Oesen et al. 2015) used the 30‐s timed sit‐to‐stand test. The data were combined as sit‐to‐stand tests and the SMD was calculated (Table 3), resulting in a forest plot (Figure 2D). The SMD was −0.18 (*p* = 0.04; 95% CI, −0.59 to 0.23). However, no clear differences between the two groups were observed. No statistical heterogeneity was observed for the sit‐to‐stand tests (*I* ^2^ = 32%, *χ* ^2^ = 4.39, *p* = 0.22).

#### 3.5.2 Gait Speed

Two studies (Chanet et al. 2017; Oesen et al. 2015) (80 individuals) provided data on gait speed. The mean difference was −0.02 (*p* = 0.84; 95% CI, −0.16 to 0.16). No clear differences between the two groups were observed (Table 3, Figure 2E). No statistical heterogeneity was observed for the gait speed (*I* ^2^ = 0%, *χ* ^2^ = 0.2, *p* = 0.65).

### 3.6 Sub‐Group Analysis in Critical and Important Outcomes

The subgroup analysis could not be conducted because included studies numbers were below 10. The sensitivity analysis was conducted for the presence or absence of frailty and the with or without of exercise interventions; there were no differences in critical outcomes and important outcomes for physical functions.

#### 3.6.1 Harms

The harms were evaluated for decreased dietary intake and renal function deterioration. Data on dietary intake were available from six studies (Tieland et al. 2012; Chanet et al. 2017; Ottestad et al. 2017; Ispoglou et al. 2016; de Azevedo Bach et al. 2022; Van Wymelbeke et al. 2016). The 249 pairs included in these studies were compared with the control group as SMD. The standard mean difference was −0.19 (*p* = 0.36; 95% CI, −0.59 to 0.22). As a result of the sensitivity analysis, one trial with a high RoB (Van Wymelbeke et al. 2016) was excluded from the analysis. The 181 pairs included in these studies were compared with the control group as SMD (Table 3), and a forest plot (Figure 2F). The SMD was −0.35 (*p* = 0.05; 95% CI, −0.70 to 0.00). Morning protein intake resulted in a marginally significant decrease in dietary food intake. No statistical heterogeneity was observed for decreased dietary intake (*I* ^2^ = 25%, *χ* ^2^ = 0.04, *p* = 0.26). Renal function before and after the intervention was reported in just one study (Van Wymelbeke et al. 2016), a meta‐analysis was not conducted.

The sensitivity analysis was conducted for with or without of exercise interventions. As a result of the sensitivity analysis, one trial with exercise intervention (de Azevedo Bach et al. 2022) was excluded from the analysis. The SMD was −0.47 (*p* = 0.01; 95% CI, −0.82 to −0.11). Morning protein intake without exercise resulted in a significant reduction in daily food intake. No statistical heterogeneity was observed for decreased daily food intake (*I* ^2^ = 11%, *χ* ^2^ = 0.01, *p* = 0.34).

## 4 Discussion

Regarding the optimal timing of protein intake for improving muscle mass, Wirth et al. (2020) reviewed young and older adults separately and concluded that protein intake is effective at any timing in a systematic review. This study conducted a meta‐analysis on the effectiveness of protein intake at breakfast in older persons and did not demonstrate significant effects on lean mass, muscle strength, or physical function.

As a minimum amount, 1.0 g/kg‐BW/day of protein combined with exercise is recommended in the guideline for sarcopenia (Sayer and Cruz‐Jentoft 2022) and frailty (Dent et al. 2019). Similarly, the ESPEN expert group recommends 1.0–1.2 g/kg‐BW/day for healthy older persons (Deutz et al. 2014). In the seven extracted RCTs, the participants in the intervention group ate an additional 11–21 g of protein for breakfast. However, it is unclear whether the recommended protein intake for body weight was met. Furthermore, a previous study (Liao et al. 2018) which is similar to the guidelines (Sayer and Cruz‐Jentoft 2022; Dent et al. 2019) has also pointed out that protein supplementation alone did not improve muscle mass or strength. This study may support this review. In included studies, none of the RCTs evaluated or excluded sarcopenia. These may also have contributed to the lack of the observed effects of protein intake at breakfast.

In this study, subgroup analysis was not performed because the number of included studies was fewer than 10. Sensitivity analyses comparing the presence versus absence of frailty and the inclusion versus exclusion of exercise interventions showed no significant differences in critical outcomes or important physical function outcomes. However, a significant reduction in daily food intake was observed in trials that did not include exercise interventions.

A high‐protein breakfast has been widely reported to enhance satiety (Blom et al. 2006; Leidy et al. 2015; Veldhorst et al. 2009a, 2009b). One proposed physiological mechanism underlying this effect involves the attenuation of appetite through a blunted ghrelin response, the “satiety” hormone (Blom et al. 2006), this appetite suppression has been suggested to influence total daily energy intake (Leidy et al. 2015; Veldhorst et al. 2009a, 2009b); however, the evidence remains inconsistent. While some studies have demonstrated a reduction in overall energy intake (Veldhorst et al. 2009a), the others have reported no significant effect (Dalgaard et al. 2024). Notably, these findings are derived from clinical trials conducted in younger adult populations. Therefore, the effects of high‐protein breakfasts on food intake and total daily energy intake in older adults remain unclear. In this study, it is suggested that high‐protein breakfasts without exercise intervention may be reducing daily food intake in older adults. Although further research is needed to determine whether this has an effect when combined with exercise.

Previous studies on younger persons suggested that maintaining consistent protein intake throughout the day was desired (Mamerow et al. 2014) because fluctuating protein intake in the three meals could affect serum protein containment. Additionally, protein intake at breakfast had been shown not only to correct these fluctuations but also to improve metabolic dynamics (Pekmez et al. 2020) and body fat levels (Ikeda et al. 2020). In addition, a recent scoping review (Khaing et al. 2025) that included younger persons reported the efficacy of protein intake at breakfast, which indicated that older persons experienced no benefits in muscle mass, muscle strength, or physical function from consuming protein at breakfast. Furthermore, although the evidence level is very low, it has been suggested that consuming proteins at breakfast may reduce total daily food intake. This point may support the result of the present meta‐analysis.

However, extracted 7 RCTs did not register the study protocols on the International Clinical Trials Registry Platform, and several studies were open‐label. Additionally, the overall number of cases was small, resulting in a relatively low rating of study quality. While our results differ from those of Wirth et al. (2020), due to limitations in study quality, further evidence is needed to determine the efficacy of protein intake at breakfast on physical function. This requires the use of larger, higher‐quality RCTs.

This review has several limitations. First, a small number of cases (274 persons) were extracted for the meta‐analysis. Further evidence accumulation is a future challenge as the detection power may have been low. Second, six of the seven RCTs were conducted in EU countries, potentially introducing racial bias and limiting the applicability of the findings to Asian or African populations. Third, many outcomes lacked consistency, and the confidence intervals in the forest plot straddled the line between easy and harmful. Forth, the possibility cannot be ruled out that frail adults and nursing home residents in included studies have also sarcopenia. In addition to the limited number of cases, the number of included studies was insufficient, highlighting the need for high‐quality studies.

## 5 Conclusions

The evidence suggests that consuming protein at breakfast results in no difference in muscle mass, muscle strength, or physical function in older adults. However, as an adverse event, increased protein intake at breakfast may reduce little on total daily food intake when not combined with exercise but the evidence is very uncertain. Due to the limited number of RCTs and cases extracted, the level of evidence is low and conclusions are limited. Therefore, future high‐quality, large‐scale RCTs are needed to investigate the effects of protein consumption at breakfast on muscle mass and strength in both healthy and frail older adults.

## Author Contributions

**Takeshi Hasegawa:** project administration, supervision, writing – original draft, writing – review and editing, funding acquisition. **Hiroyuki Ohtsuka:** data curation, investigation. **Hisashi Noma:** formal analysis, methodology, writing – original draft, writing – review and editing, funding acquisition. **Masaaki Matoba:** data curation, writing – review and editing. **Erika Ota:** writing – review and editing. **Naonori Tashiro:** data curation, investigation. **Takashi Ikeda:** conceptualization, data curation, formal analysis, investigation, methodology, project administration, writing – original draft, writing – review and editing, funding acquisition. **Noyuri Yamaji:** investigation, methodology, supervision, formal analysis.

## Funding

This work was supported by a Grant‐in‐Aid for Research in Nagoya City University (Grant Number 2512018) and Grants‐in‐Aid for Scientific Research ＜KAKENHI＞ (grant numbers: JP22H03554 and JP24K06239) from the Japan Society for the Promotion of Science.

## Ethics Statement

This systematic review and meta‐analysis was conducted on published studies; therefore, it did not involve any human or animal studies performed by the authors.

## Consent

The authors have nothing to report.

## Conflicts of Interest

The authors declare no conflicts of interest.

## Supporting information


**Table S1:** Literature search strategy for each database.

## Data Availability

Data sharing not applicable to this article as no datasets were generated or analyzed during the current study.
