# Supplementary material for: Protein or Amino Acid Intake at Breakfast for Muscle Mass Gain in Older Adults: A Systematic Review and Meta‐Analysis
Source: Food Sci Nutr. 2026 Jul 24;14(7):e72171. doi: 10.1002/fsn3.72171 (PMC13400828; doi:10.1002/fsn3.72171)
Supplement: Supplementary file 1 — Table S1: Literature search strategy for each database. [file FSN3-14-e72171-s001.docx]

**Table S1. Literature search strategy for each database**

**PubMed search strategy**

|  | Search formula | Hit No. |
| --- | --- | --- |
| #1 | Aged[mh] OR aged[tiab] OR elder*[tiab] OR older*[tiab] OR geriatr*[tiab] OR old-adult*[tiab] OR old-age*[tiab] OR senil*[tiab] OR senior*[tiab] | 4,365,170 |
| #2 | Proteins[mh] OR protein[tiab] OR proteins[tiab] OR Amino Acids[mh] OR amino acid*[tiab] OR BCAA[tiab] OR aminoisobutyric acid*[tiab] OR isoleucine*[tiab] OR alloisoleucine*[tiab] OR lysine*[tiab] OR valine*[tiab] | 8,815,337 |
| #3 | Breakfast*[tw] OR morning[tiab] | 64,843 |
| #4 | #1 AND #2 AND #3 | 3,922 |
| #5 | Controlled Clinical Trial[pt] OR randomized[tiab] OR randomised[tiab] OR placebo[tiab] OR Clinical Trials as Topic[mesh:noexp] OR randomly[tiab] OR trial[ti] NOT (Animals[mh] NOT Humans [mh]) | 1,517,400 |
| #6 | #4 AND #5 | 1,235 |

**CENTRAL search strategy**

|  | Search formula | Hit No. |
| --- | --- | --- |
| #1 | [mh Aged] OR (aged OR elder* OR older* OR geriatr* OR old-adult* OR old-age* OR senil* OR senior*):ti,ab,kw | 697,197 |
| #2 | [mh Proteins] OR [mh "Amino Acids"] OR (protein OR proteins OR amino-acid* OR BCAA OR aminoisobutyric-acid* OR isoleucine* OR alloisoleucine* OR lysine* OR valine*):ti,ab,kw | 260,111 |
| #3 | (breakfast* OR morning):ti,ab,kw | 33,695 |
| #4 | #1 AND #2 AND #3 | 2,948 |
|  | Cochrane Reviews | 2 |
|  | Trials | 2,946 |

**EMBASE search strategy**

|  | Search formula | Hit No. |
| --- | --- | --- |
| #1 | aged'/exp OR aged OR 'aged people'/exp OR 'aged people'  OR 'aged person'/exp OR 'aged person' OR 'elderly'/exp OR elder* OR 'very elderly'/exp OR 'very elderly' OR 'frail elderly'/exp OR 'frail elderly' OR 'elder people' OR 'older adults'/exp OR 'older adult*' OR 'older people'/exp OR 'older people' OR older* OR 'old adult' OR 'old age'/exp OR 'old age' OR 'senescence'/exp OR senescence OR 'senility'/exp OR senilit* OR 'senior citizen'/exp OR 'senior citizen' | 6,424,338 |
| #2 | protein'/exp OR proteins OR protein OR 'amino acid'/exp OR 'amino acid*' OR 'branched chain amino acid'/exp OR 'branched chain amino acid' OR 'bcaa' OR '2 amino 2 methylpropionic acid'/exp OR 'aminoisobutyric acid' OR 'isoleucine'/exp OR isoleucin* OR 'alloisoleucine'/exp OR alloisoleucin* OR 'lysine'/exp OR lysin* OR 'valine'/exp OR valin* | 8,748,526 |
| #3 | breakfast'/exp OR breakfast OR 'morning'/exp OR morning OR (('meal'/exp OR meal) AND (breakfast OR morning)) | 133,772 |
| #4 | #1 AND #2 AND #3 | 8,243 |
| #5 | clinical trial'/de OR 'randomized controlled trial'/de OR 'randomization'/de OR 'single blind procedure'/de OR 'double blind | 2,987,287 |
| #6 | #4 AND #5 | 2,558 |
